# PET Imaging of [^11^C]MPC-6827, a Microtubule-Based Radiotracer in Non-Human Primate Brains

**DOI:** 10.3390/molecules25102289

**Published:** 2020-05-13

**Authors:** Naresh Damuka, Paul W. Czoty, Ashley T. Davis, Michael A. Nader, Susan H. Nader, Suzanne Craft, Shannon L. Macauley, Lindsey K. Galbo, Phillip M. Epperly, Christopher T. Whitlow, April T. Davenport, Thomas J. Martin, James B. Daunais, Akiva Mintz, Kiran Kumar Solingapuram Sai

**Affiliations:** 1 Department of Radiology, Wake Forest School of Medicine, Winston-Salem, NC 27157, USA; ndamuka@wakehealth.edu (N.D.); A.Davis@wakehealth.edu (A.T.D.); mnader@wakehealth.edu (M.A.N.); cwhitlow@wakehealth.edu (C.T.W.); 2 Department of Physiology and Pharmacology, Wake Forest School of Medicine, Winston-Salem, NC 27157, USA; pczoty@wakehealth.edu (P.W.C.); snader@wakehealth.edu (S.H.N.); lgalbo@wakehealth.edu (L.K.G.); pepperly@wakehealth.edu (P.M.E.); adaven@wakehealth.edu (A.T.D.); jdaunais@wakehealth.edu (J.B.D.); 3 Department of Internal Medicine-Gerontology, Wake Forest School of Medicine, Winston-Salem, NC 27157, USA; suzcraft@wakehealth.edu (S.C.); smacaule@wakehealth.edu (S.L.M.); 4 Department of Anesthesiology, Wake Forest School of Medicine, Winston-Salem, NC 27157, USA; tjmartin@wakehealth.edu; 5 Department of Radiology, Columbia University, New York, NY 10016, USA; am4754@cumc.columbia.edu

**Keywords:** PET imaging, microtubule, blood–brain barrier, reproducibility, non-human primate

## Abstract

Dysregulation of microtubules is commonly associated with several psychiatric and neurological disorders, including addiction and Alzheimer’s disease. Imaging of microtubules in vivo using positron emission tomography (PET) could provide valuable information on their role in the development of disease pathogenesis and aid in improving therapeutic regimens. We developed [^11^C]MPC-6827, the first brain-penetrating PET radiotracer to image microtubules in vivo in the mouse brain. The aim of the present study was to assess the reproducibility of [^11^C]MPC-6827 PET imaging in non-human primate brains. Two dynamic 0–120 min PET/CT imaging scans were performed in each of four healthy male cynomolgus monkeys approximately one week apart. Time activity curves (TACs) and standard uptake values (SUVs) were determined for whole brains and specific regions of the brains and compared between the “test” and “retest” data. [^11^C]MPC-6827 showed excellent brain uptake with good pharmacokinetics in non-human primate brains, with significant correlation between the test and retest scan data (*r* = 0.77, *p* = 0.023). These initial evaluations demonstrate the high translational potential of [^11^C]MPC-6827 to image microtubules in the brain in vivo in monkey models of neurological and psychiatric diseases.

## 1. Introduction

Microtubules are complex scaffolding molecules that work closely with actin to provide structural integrity to the cell network [1]. Microtubules are believed to play a critical role in cell division and core messenger activities, and are highly regulated in terms of length, number, allocation, positioning, and confirmation [2]. When their structural integrity is compromised, dysfunction is observed in key biophysical functions including cellular signaling and axoplasmic transport, which lead to the development of neurological and psychiatric disorders. [3,4,5]. For example, the structural changes in neurons induced by alcohol contribute to the long-lasting nature of alcohol use disorder (AUD) [6,7,8]. Repeated exposure to alcohol induces structural plasticity [9,10] in brain reward circuits and changes in the density and morphology of dendritic spines, which have significant consequences including cognitive deficits [11]. Any abnormalities in these functions can lead to a variety of brain diseases, including Alzheimer’s disease (AD), multiple sclerosis, amyotrophic lateral sclerosis, psychiatric disorders, addiction, and cancer [2,12,13,14,15,16,17]. Importantly, hyperphosphorylation of tau, a microtubule-associated protein, leads to the development of major neurodegenerative diseases including AD and other AD-related disorders (ADRDs) [16,18,19,20]. However, microtubule structure and functions are sufficiently complex that the sequence of events from microtubule disruption to the expression of disease largely remains unknown [1,7,21,22].

**Figure 1:**
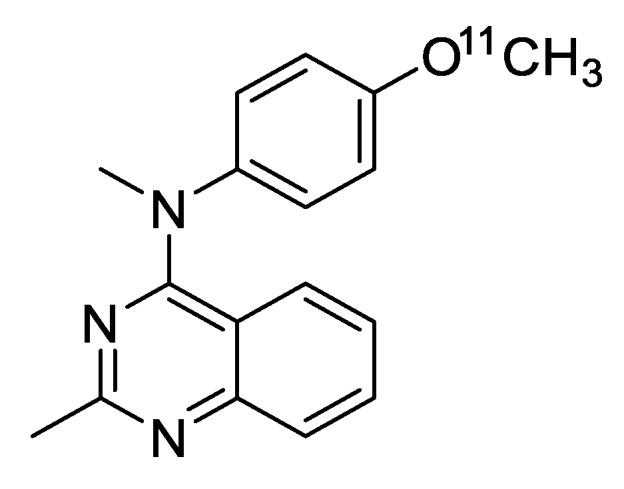
Structure of [^11^C]MPC-6827.

Quantification of microtubules in vivo using PET (positron emission tomography) can provide significant information about altered disease mechanisms and can also be used evaluate therapeutic strategies to monitor the progress of treatment [8,23,24]. We screened multiple microtubule-targeting agents (MTAs) as potential PET ligands and identified MPC-6827 as our lead candidate [25]. MPC-6827 was a well-characterized MTA with high affinity for the β-tubulin site (IC_50_ = 1.5 nM) [25]. It has been proven safe in human subjects, with ideal pharmacokinetics, and has undergone multiple clinical trials for the treatment of glioblastoma and other advanced cancers [25,26]. Our laboratory recently reported the design, development, and in vivo evaluations of the first brain-penetrating microtubule-based PET radiotracer, [^11^C]MPC-6827 (Figure 1) in rodents [27]. Microtubule-based PET imaging could provide a novel imaging biomarker platform with which to diagnose disease pathology early on, and we are currently working on evaluating the microtubule-based PET imaging properties of [^11^C]MPC-6827 in murine models of AD [28]. To explore the translational potential of [^11^C]MPC-6827, non-human primate (NHP) brain PET imaging was initially performed in two adult male rhesus monkeys [29], in which we demonstrated high uptake in the whole brain. In the present study, we extended the characterization of [^11^C]MPC-6827 to include brain measures of between-subject variability and within-subject “test–retest” variability in four adult male cynomolgus monkeys.

## 2. Results and Discussion

**Figure 2:**
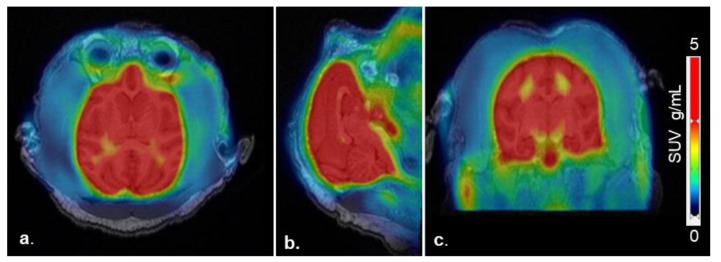
Representative (**a**). axial, (**b**). sagittal, and (**c**). coronal PET-MR coregistered images from “test” and “retest” scans (*n* = 8) following an i.v. injection of [^11^C]MPC-6827 in male cynomolgus monkeys (*n* = 4).

**Figure 3:**
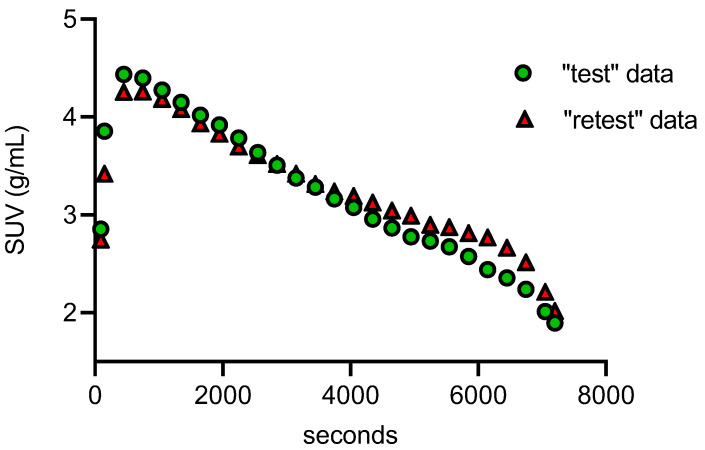
Representative “test” and “retest” whole brain time activity curves (TACs) from dynamic 0–2 h PET images (*n* = 8) from four male monkeys injected with 0.37 ± 0.03 Gbq of [^11^C]MPC-6827.

PET-MR images (Figure 2) demonstrated high brain uptake of the radiotracer. Uptake in the whole brain and specific regions of the brain including caudate nucleus, putamen, globus pallidus, subthalamic nucleus, paranigral nucleus, substantia nigra, amygdala, occipital cortex, hippocampus, and cerebellum were defined by their standardized uptake value (SUV), calculated by dividing the tracer concentration in each pixel by the injected dose per body mass. These data were used to generate time activity curves (TACs) [30] (Figure 3) using the PMOD NEURO (Ver 3.5, PMOD Technologies LLC, Zurich, Switzerland) software analysis tool [31].

**Figure 4:**
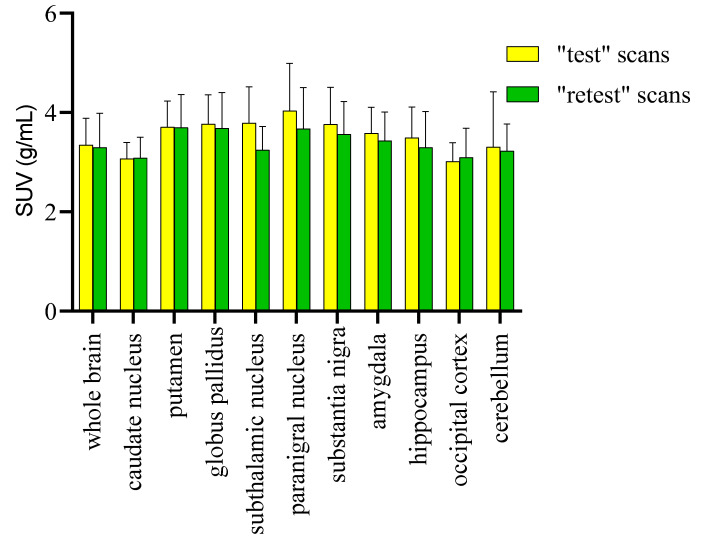
Representative standard uptake values (SUVs) from different regions of brain obtained from dynamic 0–2 h PET scans from male monkeys (*n* = 4) injected with 0.37 ± 0.03 Gbq of [^11^C]MPC-6827. The data are expressed in mean SUVs (±SEM) g/mL units.

The test–retest scans did not differ significantly regarding the injected dose (0.37 ± 0.05 GBq), specific activity (140.5 ± 3.7 GBq/µmol), and radiochemical purity (99.0 ± 0.5%). No significant differences in body weight (8.2 ± 1.0 kg) or vital signs were found between the test and retest scans. PET imaging data were presented as the mean SUV ± SD from eight PET/CT scans (four test and four retest scans). The significant differences among multiple groups were calculated by one-way analysis of variance and *p* values ≤ 0.05 were considered statistically significant. From the whole-brain TACs (Figure 3), the radiotracer was injected over a period of 45–60 sec and the concentration of [^11^C]MPC-6827 peaked at ~ 4.0–4.5 min after the intravenous tracer injection in all the scans (*n* = 8). Radioactive uptake was slightly higher in paranigral nucleus, putamen, and globus pallidus (SUV_mean_ = 3.81 ± 0.41 g/mL) compared to caudate nucleus and occipital cortex (SUV_mean_ = 3.01 ± 0.31 g/mL) (Figure 4). The mean SUVs (g/mL) for whole brain for all the test and retest scans were between 3.34 ± 0.22 and 3.58 ± 0.12, with a variation of < 1%. The reproducibility of [^11^C]MPC-6827 PET scans with respect to SUV measurements was calculated using relative difference % (rel diff %) and absolute variability % (abs var %) between the test and retest scans. Rel diff % was calculated as ((retest SUV − test SUV)/test SUV) × 100 and abs var % as ((retest SUV − test SUV)/((retest SUV + test SUV)/2) × 100. With respect to reproducibility evaluation [30], the relative difference of SUVs for whole brain and the organs of interests between the “test” and “retest” scans varied from −0.3% to −14%, and the absolute variability between the same parameters/regions from “test” and “retest” was < 0.5%. The intra-subject repeatability [30] expressed as the coefficient of variance in test scans showed an average of 16.78% ± 4.02% and in “retest” scans of 18.03% ± 3.01%. When a two-way ANOVA (Sidak’s comparison test, GraphPad Prism 7.05) was performed with SUV_mean_ values of whole brain and the brain regions between the test scans (test-1 and test-2), there was no statistical significance (*p* > 0.999). Consistent with the lack of significance between the two tests, the correlation between the “test” and “retest” was statistically significant, *p* = 0.023, with correlation coefficient (*r*) = 0.77, standard error of mean (SEM) = 0.053 and confidence interval (95%) between −0.26 to −0.012.

## 3. Material and Methods

[^11^C]MPC-6827 was produced following our previously reported radiochemistry methods [27,32]. Briefly base-assisted [^11^C]MeI methylation of desmethyl MPC-6827 in NaOH/DMF at 80 °C for 5 min followed by HPLC and C18 SepPak trapping and elution resulted in [^11^C]MPC-6827 with high-quality specifications, i.e., > 98% radiochemical purity, 43% radiochemical yield, and ~3890–4500 mCi/µmol specific activity, decay corrected to end of synthesis (EOS).

PET imaging of [^11^C]MPC-6827 was performed in adult male cynomolgus monkeys (*n* = 4, ~ 8.2 ± 1.0 kg) and the same cohort was rescanned with PET using [^11^C]MPC-6827 after ~7 days. All animal housing and handling and all experimental procedures were performed in accordance with the National Institute of Health Guide for the Care and Use of Laboratory Animals (2011) and were approved by the Animal Care and Use Committee (ACUC) of Wake Forest University. Environmental enrichment was provided as outlined in the ACUC of Wake Forest University Non-Human Primate Environmental Enrichment Plan. Monkeys were fasted overnight before the PET study. The monkeys were initially anesthetized using ketamine (10 mg/kg, i.m.) and transported to the PET scanner suite. Isoflurane (3–5%) was administered via nose cone until each monkey was intubated with an endotracheal tube and isoflurane anesthesia was maintained at 1.5% isoflurane/oxygen throughout the PET scanning procedure. NHPs were placed in the scanner and a catheter was inserted into an external saphenous vein for tracer injection and fluid replacement. Body temperature was maintained at 40 °C with a water-circulating heating pad, and vital signs including heart rate, blood pressure, respiration rate, and temperature were monitored throughout the scanning procedure.

First, an initial low-dose CT-based attenuation correction scan was acquired. Next, all monkeys received an intravenous dose of 0.37 ± 0.03 GBq [^11^C]MPC-6827 and 0–120 min dynamic brain PET scans were acquired using a 64 slice GE PET/CT discovery scanner [33]. For each frame, image reconstruction of the acquired emission data was done with full quantitative corrections including attenuation and reconstructed into 2 × 30 sec, 3 × 1 min, 5 × 2 min, 4 × 4 min, and 9 × 10 min frames [34,35]. Because certain brain regions are more affected than others during progression of addiction and AD [24,31], we selected those commonly studied/affected areas of brain for our image analyses [36]. To identify these regions, anatomical images were acquired for all the monkeys using magnetic resonance imaging (MRI). Anesthesia was maintained during the scanning procedure with ketamine (15 mg/kg i.m.) and 3D MRI brain images were acquired with 3T GE signa NR scanner using typical NHP brain parameters (TE 5, TR 45, flip angle 45, RBW 15.6 kHz, FOV 18 cm, 256 × 192 matrix, slice thickness 2 mm) [37,38]. T1-weighted whole brain images were used to anatomically define spherical regions of interest (ROIs). ROIs had ~2.5 mm radii for all regions except the cerebellum, which was a 4.0 mm radius. PET images were coregistered with MRI and fused PET/MR data were analyzed using PMOD Biomedical Image Quantification Software (version 3.5; PMOD Technologies) [24,31,39].

## 4. Conclusions

The observed high correlation of [^11^C]MPC-6827 PET/CT images between the “test” and “retest” scans in NHP brains further supports the high translational potential of the radiotracer. Importantly, high SUV values in selective brain areas including putamen, globus pallidus, cortex, and hippocampus warrants additional studies to investigate microtubule regulation in models of diseases such as AUD and AD. Further studies will include [^11^C]MPC-6827 PET imaging in NHP models of substance abuse disorder, including alcohol and cocaine self-administration [39,40], and NHP model of AD [38]. We will also characterize the metabolite analyses, arterial input functions, and specificity studies, and correlate the data with PET images using sophisticated pharmacokinetic modeling [31].
